# Clinical pharmacodynamic factors in docetaxel toxicity

**DOI:** 10.1038/sj.bjc.6603872

**Published:** 2007-06-26

**Authors:** F Puisset, J Alexandre, J-M Treluyer, V Raoul, H Roché, F Goldwasser, E Chatelut

**Affiliations:** 1 Université de Toulouse et Institut Claudius-Regaud, EA3035, F-31052 Toulouse, France; 2 Faculté de Médecine Université Paris Descartes, Paris, France

**Keywords:** pharmacokinetics, pharmacodynamics, docetaxel, population analysis, individual dosing

## Abstract

Neutropenia is the main dose-limiting toxicity occurring in docetaxel treatment. The objective of this study was to identify pharmacodynamic (PD) factors responsible for the neutropaenia caused by docetaxel. Data were obtained from 92 patients treated with docetaxel as a monochemotherapy in two different treatment centres. A semiphysiological population pharmacokinetic–pharmacodynamic (PK/PD) model was applied to describe the time course of neutrophils and the neutropaenic effect of docetaxel. The plasma docetaxel concentration was assumed to inhibit the proliferation of neutrophil precursors through a linear model: Drug effect=Slope × Conc. Slope corresponds to the patients’ sensitivity to the neutropaenic effect of docetaxel. Covariate analysis was performed by testing the relationship between the patients’ characteristics and Slope using the program NONMEM. The neutropaenic effect of docetaxel showed a high interindividual variability. Three significant PD covariates were identified: serum *α*1-acid glycoprotein levels (AAG), level of chemotherapy pretreatment, and treatment centre. Extensive pretreatment was associated with an increase in Slope values meaning a higher haematotoxicity. An increase in AAG was associated with a decrease of both Slope and docetaxel plasma clearance. Patients treated in one centre had both higher Slope and docetaxel clearance. The centre effect (most likely due to a bias in the PK part of the study between the two centres) reveals the robustness of the PK/PD model. Individual dosing of docetaxel should be based on previous chemotherapy but not on the AAG level since it has a similar influence on PD and PK docetaxel parameters. This methodology should be applied to further investigate elderly patients and to identify more precisely the characteristics of previous chemotherapy that contribute to the cumulative myelotoxicity.

Neutropenia is the main dose-limiting toxicity of docetaxel treatment. This toxicity may be life-threatening, and often imposes a delay on subsequent administration that affects treatment efficacy. Altered pharmacokinetics was identified as a factor involved in the toxicity. A relationship has been observed between systemic exposure to docetaxel, as measured by the AUC, and neutropenia (Baker *et al*, 2006). Pharmacokinetic studies performed by Bruno *et al* (1996) based on a population pharmacokinetic (PK) analysis of data obtained during the clinical development of docetaxel showed that docetaxel clearance (CL) was related to hepatic function and body surface area (BSA), thus justifying measuring BSA and adjusting the dose in patients with elevated alkaline phosphatase and transaminases levels. The effect of age on CL was statistically significant but slight: a 6.7% decrease in mean CL in patients aged from 56 to 71 years. Minami *et al* (2004) did not observe any consistent difference in CL between elderly (⩾75 years) and non-elderly patients. However, they concluded that elderly patients were more sensitive to docetaxel exposure than the younger patients. More generally, evidence of interindividual variability in docetaxel haematotoxicity can be seen from all these pharmacokinetic–pharmacodynamic (PK/PD) studies: patients with similar AUC may have different neutrophil counts at nadir.

The current study describes population PK/PD analyses based on data from 92 patients included in two studies of single-agent docetaxel treatment. The analyses utilise a semimechanistic model (Figure 1) proposed by Friberg *et al* (2002), describing the effect of cytotoxic drugs on bone marrow production. After the drug administration, at any time, the cytotoxic effect of the drug (*E*_drug_) on the proliferating neutrophil cells is proportional to the plasma drug concentration (Conc) according to the equation: *E*_drug_=Slope × Conc. Slope corresponds to the patient sensitivity to the neutropaenic effect of docetaxel. Its value is subject to interindividual variability. The objective of this PK/PD analysis was to identify pharmacodynamic factors (PD covariates) involved in docetaxel neutropenia. That was performed by integrating patients’ characteristics into the model and evaluating their contribution to the interindividual variability in Slope values. The advantage of this model was the consideration of the whole concentration–time profile rather than a single PK variable such as AUC.

## MATERIALS AND METHODS

### Patients, drug treatment, and haematological evaluation

Data were obtained from 92 patients who were included in two separate clinical trials. The first one was carried out in the Institut Claudius-Regaud of Toulouse and was designed to correlate docetaxel CL to dexamethasone clearance to use as a probe of docetaxel elimination (*n*=37) (Puisset *et al*, 2007). The second trial (*n*=55) was performed in the Hôpital Cochin (Paris) to study the relationship between docetaxel toxicity and genetic polymorphisms (Tran *et al*, 2006). The two protocols were approved by their regional ethical committees. All patients provided written informed consent before enrolment in the study. Patients’ characteristics are summarised in Table 1. In both studies, docetaxel (Taxotere®, Aventis Pharma, Paris, France) was administered intravenously as a monotherapy over 1 h, at doses ranging from 70 to 100 mg m^−2^. All patients received an antiallergic and antiemetic premedication. Complete blood cell counts were performed weekly after the first cycle. No patient received any prophylactic granulocyte colony-stimulating factor.

### PK sampling and assay

Blood samples were collected before infusion, 0.5, 2, and 6 h after the end of infusion for the Toulouse Centre; before infusion, at the end of infusion, and 6 h after the end of infusion for the Paris Centre. The plasma docetaxel concentrations were measured by reverse-phase HPLC using UV absorbance detection, according to previously described methods (Puisset *et al*, 2004; Tran *et al*, 2006). The coefficients of variation for interday reproducibility and precision were lower than 15.7 and 6.0% for the analyses performed in Toulouse and Paris, respectively.

### PK/PD analyses

Data were analysed according to a nonlinear mixed effects population approach using NONMEM (Beal and Sheiner, 1982) (version V, level 1.1; GloboMax, Hanover, MD, USA). First, a PK analysis of docetaxel plasma concentrations *vs* time was performed in order to obtain docetaxel PK parameters (POSTHOC values) and a complete concentration-*vs*-time profile for each patient. Secondly, the PK/PD analysis of ANC *vs* time was performed using these individual PK parameters.

### PK analysis

Individual-specific PK parameters were obtained by analysis of docetaxel plasma concentrations *vs* time using the Bayesian estimation method previously proposed by Baille *et al* (1997). Complete plasma docetaxel concentration-*vs*-time profiles used for the PK/PD analysis were generated from this analysis. Moreover, the relationships between the covariates and plasma docetaxel CL were evaluated. Seven covariates were tested: gender, AGE, BSA, serum *α*1-acid glycoprotein level (AAG), treatment centre (CEN=0 or =1 if data corresponded to Paris or Toulouse, respectively), pretreatment PTT1 (PTT1=0 or =1 if patients underwent their first line of chemotherapy or not, respectively), and PTT2 (PTT2=0 or =1 if patients had less than two lines, or at least two lines of chemotherapy before docetaxel, respectively). The FOCE method was implemented for the estimation of the different parameters.

### PK/PD model

**Figure 1:**
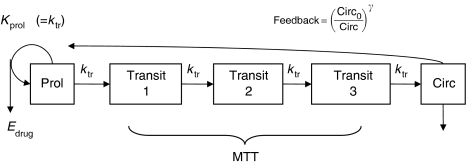
Pharmacokinetic–pharmacodynamic model describing myelosuppression following docetaxel administration (modified from Friberg *et al*, 2002). *k*_prol_, rate of cell division; *k*_tr_, rate of transit between compartments; *k*_circ_, rate of physiological elimination of circulating cells; Circ_0_/Circ, baseline circulating cell counts before drug administration/circulating blood cell count at a given time; Prol, compartment representing progenitor cells; *E*_drug_, cell loss, function proportional to docetaxel concentration in central compartment; MTT, mean transit time through the maturation delay chain.

The PD model was that proposed by Friberg *et al* (2002) for docetaxel (Figure 1). The model mimics the maturation chain of neutrophils and was based on five compartments: one compartment that represented progenitor cells [Prol], three maturation compartments [Transit], and a compartment of observed circulating neutrophils [Circ]. The generation of new cells in [Prol] was dependent on the number of cells in the compartment, that is, self-renewal or mitosis. A proliferation rate constant determining the rate of cell division (*k*_prol_), and a feedback mechanism, was modelled as (Circ_0_/Circ)^*γ*^ where Circ was the circulating blood cell count at a given time and Circ_0_ the baseline circulating cell counts before docetaxel administration. As in the original model, the rate constants were assumed to be equal: *k*_prol_, *k*_tr_, and *k*_circ_ for the rate of cell division, of the transit between compartments, and the physiological elimination of circulating cells, respectively. Three system-related parameters were estimated by NONMEM analysis: Circ_0_, MTT as the mean transit time through the maturation delay chain, and *γ* as the power factor for the feedback mechanism. Docetaxel concentration in the central compartment (Conc, corresponding to the plasma concentration) is assumed to induce cell loss from [Prol]. The drug effect, *E*_drug_, was proportional to Conc: *E*_drug_=Slope × Conc. The interindividual variability was estimated for Circ_0_, MTT, and Slope according to an exponential model. A log-normal distribution of the parameters was assumed. A HYBRID method (FOCE method for Circ_0_, FO method for MTT and Slope) was applied for the estimation of parameters. A proportional model for residual variability was used.

### Relationships between covariates and PD parameters

The analysis of the PD covariate effect was limited to the Slope parameter. Seven covariates were evaluated: gender, AGE, BSA, AAG, CEN, PTT1, and PTT2. For continuous covariates, the impact of each covariate on the Slope value was tested according to the following equation using, for example, AGE : TVSlope=*θ*_1_(AGE/mean AGE)^*θ*_2_^ where *θ*_1_ is the typical value of Slope (TVSlope) for a patient with the mean covariate value, and *θ*_2_ is the estimated influential factor for the covariate AGE. Dichotomous covariates (gender, centre, and pretreatment) were tested according to the equation using, for example, PTT: TVSlope=*θ*_1_ × *θ*_2_^PTT^ with PTT=1 or =0 for patients pretreated or not pretreated. Full and reduced models (one parameter less) were compared by the *χ*^2^ test of difference between their respective objective function values (OBJ). OBJ is equal to minus twice the log likelihood of the data. For the forward search, each covariate was univariately introduced into the basic model. A covariate was considered as significant if it decreased the OBJ by at least 3.84 (*P*<0.05, 1 degree of freedom). An intermediate model including all significant covariates was then obtained. Therefore, a stepwise backward elimination procedure was carried out. Deletion of the covariate from the intermediate model should be associated with an increase of the OBJ of at least 10.83 (*P*<0.001, 1 degree of freedom) in order to consider this covariate as relevant. Moreover, 95% confidence intervals (95% CIs) corresponding to the estimated influential factor (*θ*_2_) were calculated from the standard errors estimated by NONMEM. At each step of the covariate analysis, the 0 value should be outside the 95% CI in order to consider the covariate as significant.

## RESULTS

### PK analysis

There was a good agreement between observed plasma docetaxel concentrations and those estimated by the Bayesian method (regression line: *y*=1.002*x*+0.04, with *R*^2^=0.99), indicating that individual docetaxel plasma profiles were adequately generated. Mean (range) plasma docetaxel clearance and AUC were 40.0 (15.9–74.4) l h^−1^ and 4.1 (1.9–8.7) mg h l^−1^, respectively. An increase in AAG levels was associated with a decrease in CL, and patients treated in Toulouse had higher CLs than those treated in Paris. Multivariate analysis led to the following PK covariate model (±95%): 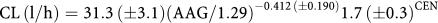 with CEN=0 or =1 if data corresponded to Paris or Toulouse, respectively.

### PD model and analysis of covariates

**Figure 2:**
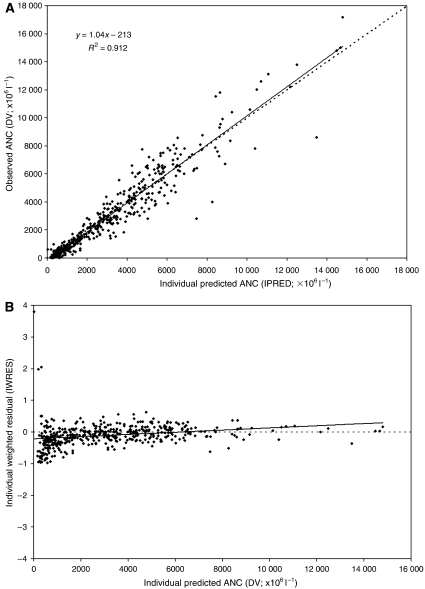
Observed neutrophil (ANC) values (**A**) or individual weighted residual (**B**) *vs* individual predicted values (- - - , identity line; —, regression line).

**Figure 3:**
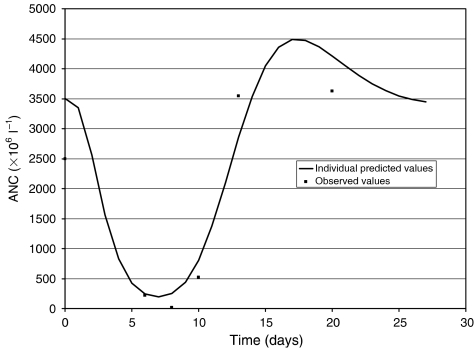
Individual predicted and observed neutrophil time course of a ‘typical’ patient.

**Figure 4:**
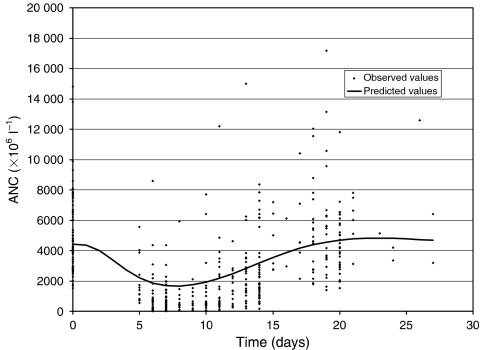
All observed ANC values and population-predicted ANC–time profile for a patient receiving a mean dose of 150 mg (85 mg m^−2^ for a patient with a BSA of 1.77 m^2^).

The structural model described well the time course of ANC after docetaxel administration (Figure 2A and B). Figure 3 shows predicted and observed neutrophil counts for an individual selected on the basis of the magnitude of his typical residual error (average absolute weighted residual is similar to the population mean value, i.e., 36%). The mean parameters of the PD model and their corresponding interindividual variability are shown in Table 2. Values previously obtained for docetaxel by Friberg *et al* (2002) are indicated for comparison. Figure 4 shows all the observed ANC values and population-predicted ANC–time profile (i.e., PRED *vs* time corresponding to the mean system-related parameters and Slope values following the mean docetaxel dose of 150 mg).

### Analysis of covariates

**Figure 5:**
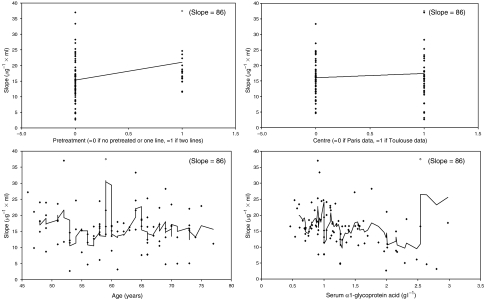
Individual predicted Slope values *vs* age, *α*1-acid glycoprotein, pretreatment, and treatment centre.

**Figure 6:**
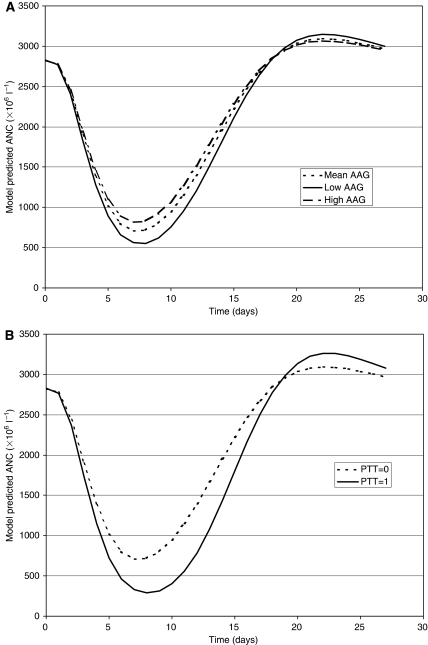
Effect of covariates on the time course of the neutropenic effect of docetaxel: ±1 s.d. from the mean *α*1-acid glycoprotein value (**A**), and pretreatment with PTT=1 for patients heavily pretreated (**B**).

During the individual testing, four covariates (i.e., AGE, PTT2, AAG, and CEN) were significantly correlated with Slope. Figure 5 shows the individual Slope values *vs* each of these covariates. The intermediate model was based on these four covariates. The absolute *θ* value corresponding to AGE was not significantly different from 0. The final covariate model was based on PTT2, AAG, and CEN since their corresponding CIs excluded the 0 value, and deletion of each of them was associated with a significant increase of the OBJ. The relationship between the Slope values and the covariates is shown in Table 3. Docetaxel sensitivity (Slope values) was increased overall by 69% in patients previously treated by at least two previous chemotherapy regimens (PTT2). The figure for patients treated at the Toulouse Centre was 82%, and was nearly inversely proportional to the AAG level. The effect of AAG and PTT2 on the ANC profile is shown in Figure 6 and shows that PTT2 had a real impact on the ANC count but AAG did not due to its simultaneous effect on the PK of docetaxel.

## DISCUSSION

The semimechanistic PK/PD model proposed by Friberg *et al* (2002) described well the ANC values observed after docetaxel administrations in our patient population. The typical values corresponding to the system-based PD parameters (Circ_0_, MTT, and *γ*) and those related to the drug effect (Slope) were consistent with the values previously obtained for docetaxel. Goodness-of-fit for the PK/PD model is illustrated by the agreement between the observed values of ANC and the predicted individual values (Figure 2), and the representative example of the data from a single patient (Figure 3). The patient is also representative of a limitation of the model: the lowest ANC values (below 100 × 10^6^ l^−1^) were poorly adjusted to the individual predicted values that were higher than the observed values. However, these predicted ANC values were within the 0–500 × 10^6^ l^−1^ range (corresponding to grade 4 haematological toxicity), which is considered as clinically critical. The main advantage of this approach was to allow us to integrate data from patients with sparse ANC values. The normal evaluation of haematological toxicity (i.e., based on the nadir value) cannot be carried out when some post-chemotherapy ANC counts are missing.

An unexpected result was that the patients’ sensitivity was significantly higher (+82% in Slope values) in patients treated in the Toulouse Centre (CEN=1) than those treated in Paris (CEN=0). This PK/PD result should be analysed together with that of the PK analysis: docetaxel CL was also higher (+70%) for CEN=1. We may hypothesise that differences at the analytical level (i.e., HPLC procedure) between the two centres were responsible for a bias in the determination of docetaxel CL. Since the plasma docetaxel levels seemed to have been underestimated in Toulouse compared with those determined in Paris, the PK/PD analysis relating cytotoxic effects (*E*_drug_) to those docetaxel plasma levels led to higher Slope values for the Toulouse data. The difference in blood sampling between the two centres may also be responsible of an overestimation of the individual docetaxel CL in Toulouse in comparison with those determined in Paris, generating this apparent difference in Slope values. Rather than revealing a clinically pertinent covariate, these results show the robustness of the PK/PD model: the PK/PD analysis corrects a bias at the PK level.

Slope was significantly dependent on the patient's pretreatment. Patients who received at least two treatments of chemotherapy before docetaxel had a value almost twice as high as those not pretreated or who had previously received only one line of chemotherapy. The term 1.69^PTT2^ from the final covariates model (Table 3) suggests that the dose of docetaxel used as a third line should be reduced by about 40% from the usual dose to give the same degree of neutropaenia. This result indicates that the bone marrow reserve of the patients seems to be affected by the number of previous chemotherapy treatments. We may anticipate that the neutropaenic effect of docetaxel is also dependent on the quality and the extent of the prior chemotherapy. A more precise description of the myelotoxic aspects of the chemotherapy would have improved the model, but subdivision of the data set to accommodate these additional descriptions of the prior chemotherapy would require a larger number of patients.

The baseline AAG level was the other significant covariate. Increased AAG resulted in a decreased Slope value. This relationship was also expected since docetaxel is extensively bound to AAG, and this is mainly responsible for interindividual variability in docetaxel unbound fraction (*f*_u_) (Urien *et al*, 1996). In the present NONMEM analysis, the Slope values link total plasma docetaxel concentrations and neutropaenic responses. That is why, between two patients with equal total plasma docetaxel concentrations, the one with higher *f*_u_ value due to a lower AAG level, will have a higher Slope value. By an original and semimechanistic-based approach, these results confirm those obtained by Baker *et al* (2005) by a two-stage approach: the percentage decrements in ANC were better correlated with the AUC of unbound docetaxel than with that of total docetaxel. However, the relationship between the Slope values and the AAG level does not lead to dosing recommendations based on the AAG level. Indeed, several studies have confirmed that the docetaxel CL is dependent on *f*_u_, with a lower CL in patients with high AAG levels (Urien *et al*, 1996; Baker *et al*, 2005). Interindividual variability in AAG is not associated with marked changes in the AUC of free docetaxel. As illustrated by Figure 6A, the increase in AAG was associated with only a modest decrease in toxicity since it was also associated with a decrease in docetaxel CL. All these results suggest that dose should not be individualised according to baseline AAG level, but that protein binding phenomena should be considered when the relationship between total plasma docetaxel concentrations and PD is evaluated.

As previously mentioned in the introduction, several authors have suggested that elderly patients experienced more profound docetaxel myelotoxicity (Minami *et al*, 2004; ten Tije *et al*, 2005). On the contrary, our PK/PD analysis showed that the Slope values did not increase with age, as the plot of Slope values against age (Figure 5) did not show any trend. However, it should be noted that all but two of our patients were younger than 75 years.

In conclusion, this analysis enabled us to identify the chemotherapy administered before docetaxel treatment as a significant PD factor in haematotoxicity. These results suggest that the lower dose of docetaxel for treatment of prostate cancer (i.e., 75 mg m^−2^) *vs* breast cancer (100 mg m^−2^) remains empirical since gender was not a factor contributing to a greater sensitivity. More generally, it illustrated the potential use of PK/PD models to identify subgroups of patients with a higher risk of toxicity. In particular, this methodology should be applied to further explore the haematotoxicity of docetaxel in older patients and to identify more precisely the characteristics of the previous chemotherapy that contribute to the cumulative myelotoxicity.

## Figures and Tables

**Table 1:** Patient characteristics: comparison between centres

	**All patients *n*=92**	**Toulouse *n*=37**	**Paris *n*=55**	
**Patients characteristics**	**Mean (range)**	**Mean (range)**	**Mean (range)**	***P*-values**
Age (years)	60.7 (46–77)	60 (46–77)	61 (47–75)	NS
Body weight (kg)	70.8 (39–106)	70.9 (49–106)	70.7 (39–100)	NS
Body surface area (m^2^)^a^	1.77 (1.35–2.20)	1.78 (1.46–2.10)	1.77 (1.35–2.20)	NS
*α*1-Acid glycoprotein (g l^−1^)	1.29 (0.46–2.98)	1.51 (0.57–2.98)	1.13 (0.46–2.64)	<0.01
Serum albumin (g l^−1^)	36 (16–45)	35 (23–43)	37 (16–45)	NS
				
	Number	Number	Number	
AST: >1.5 ULN/<1.5 ULN	12/80	8/29	4/51	<0.01
ALT: >1.5 ULN/<1.5 ULN	8/84	5/32	2/53	<0.01
Gender (male/female)	47/45	19/18	28/27	NS
Number of lines of previous chemotherapy treatments: 0/1/2 or >2	27/44/21	10/19/8	17/25/13	NS
Performance status: 0/1/2/3	13/61/16/2	8/25/3/1	5/36/13/1	<0.01
Docetaxel doses: 70/75/85/100 mg m^−2^	1/29/39/23	0/21/0/16	1/8/39/7	<0.001
Primary disease: breast/prostate/lung/others	31/27/15/19	15/12/2/8	16/15/13/11	NS

ALT=serum alanine amino transferase; AST=serum aspartate amino transferase; NS=nonsignificant; ULN=upper limit of normal.

a Calculated according to the Dubois formula.

**Table 2:** Mean parameters and IIV corresponding to the structural pharmacodynamic model

**Study**	**Present study**		**Friberg *et al* (2002)**	
**parameters**	**Estimate**	**RSE (%)**	**Estimate**	**RSE (%)**
Circ_0_ (10^9^ l^−1^)	4.41	6.3	5.05	1.9
IIV Circ_0_ (%)	49.2	18.3	42	7
MTT (h)	96.2	8.8	88.7	2.5
IIV MTT (%)	25.5	40.2	16	24
*γ*	0.146	42.5	0.161	3.7
Slope (*μ*g^−1^ ml)	7.76	7.4	10.6	5.2
IIV Slope (%)	96.5	42	60	14
Proportional residual error (%)	35.6	21.3	27.3	—
Additive residual error (ANC 10^9^ l^−1^)	Not applicable	—	1.15	—

Circ_0_=baseline circulating cell counts before drug administration; *γ*=power factor for the feedback mechanism; IIV=interindividual variability; MTT=mean transit time through the maturation delay chain; RSE=relative standard error related to the corresponding variance term; Slope=drug sensitivity.

Values of the present study and those previously obtained by Friberg *et al* (2002) for docetaxel.

**Table 3:** Relationship between docetaxel neutropaenic sensitivity (Slope in *μ*g^−1^ ml) and patients’ covariates

**Models**	**Mean (±95% CI)**	**IIV Slope (%)**	**ΔOBJ^a^**	***P*-values**
*Intermediate model*				
Slope=*θ*_1_(AGE/60.7)^*θ*_2_^(AAG/1.29)^*θ*_3_^ *θ*_4_^PTT2^*θ*_5_^CEN^	*θ*_1_=7.91 (±1.64) *θ*_2_=−0.68 (±1.01) *θ*_3_=−0.74 (±0.18)*θ*_4_=1.48 (±0.45) *θ*_5_=1.77 (±0.49)	44	−5	NS
				
*Final model*				
Slope=*θ*_1_(AGE/1.29)^*θ*_2_^*θ*_3_^PTT2^*θ*_4_^CEN^	*θ*_1_=7.40 (±1.22) *θ*_2_=−0.72 (±0.18) *θ*_3_=1.69 (±0.32) *θ*_4_=1.82 (±0.46)	44	—	—
				
*Alternative models*				
Slope=*θ*_1_(AAG/1.29)^*θ*_2_^*θ*_3_^CEN^	*θ*_1_=7.73 (±1.45) *θ*_2_=−0.80 (±0.27) *θ*_3_=1.88 (±0.62)	52	+39	<0.001
Slope=*θ*_1_(AAG/1.29)^*θ*_2_^*θ*_3_^PTT2^	*θ*_1_=7.86 (±1.39) *θ*_2_=−0.51 (±0.27) *θ*_3_=−1.94 (±0.58)	59	+46	<0.001
Slope=*θ*_1_*θ*_2_^PTT2^*θ*_3_^CEN^	*θ*_1_=7.5 (±1.4) *θ*_2_=1.42 (±0.69) *θ*_3_=2.15 (±0.75)	57	+71	<0.001

AAG=serum *α*1-acid glycoprotein level; CEN=0 or=1 if data corresponded to Paris or Toulouse, respectively; CI=confidence interval; IIV=interindividual variability; NS=nonsignificant; PTT2=0 or=1 if patient received less than two lines, or at least two lines of chemotherapy before docetaxel, respectively.

a Difference in objective function value in comparison with final covariate model.
